# Supplementary material for: Immunisation with Transgenic L. tarentolae Expressing Gamma Glutamyl Cysteine Synthetase from Pathogenic Leishmania Species Protected against L. major and L. donovani Infection in a Murine Model
Source: Microorganisms. 2023 May 17;11(5):1322. doi: 10.3390/microorganisms11051322 (PMC10223578; doi:10.3390/microorganisms11051322)
Supplement: Supplementary file 1 [file microorganisms-11-01322-s001.zip › microorganisms-2296211-supplementary.pdf]

Table of contents for supplementary data

	Page No
1. Figure S1. Schematic to generate the pSSUMCS intermediate plasmid for producing *L. tarentolae* promastigotes expressing γGCS from *L. donovani, L. major,* or *L. mexicana*.	3
2. Figure S2. Schematic to generate the pTHGFPGCS intermediate plasmids used for producing *L. tarentolae* promastigotes expressing γGCS from *L. donovani, L. major,* or *L. mexicana*.	4
3. Figure S3. Schematic to generate pSSUGFPGCSHis plasmids used for producing *L. tarentolae* promastigotes expressing γGCS from *L. donovani, L. major,* and *L. mexicana*.	5
4. Figure S4. Schematic to generate the deletion constructs for *L. tarentolae* γGCS.	6
**5.** Table S1. The effect of vaccination on mean change in footpad size (mm ± SD) and mean number of amastigotes present in the footpad of *L. major* infected mice.	7
6. Figure S5. The effect of vaccination on the specific antibody response of mice of *L. major* infected mice.	8
7. Figure S6. The effect of vaccination on the IL-5 (A) and IL-10 (B) production by splenocytes from control or vaccinated mice infected with *L. major*.	9
8. Figure S7. The effect of vaccination on the mean IFN-γ production of popliteal lymph node cells (A) or spleen (B) from *L. major* infected mice shown in Table S1.	10
9. Figure S8. The effect of vaccination on the mean IL-10 production of popliteal lymph node cells (A) or spleen (B) from *L. major* infected mice shown in Table S1.	11
10. Figure S9. The effect of vaccination on the mean nitrite production of popliteal lymph node cells (A) or spleen (B) from *L. major* infected mice shown in Table S1	12
11. Figure S10. The effect of vaccination with different vaccines on neutrophil influx, antibody responses and parasite burdens of mice infected with *L. donovani*.	13
12. Figure S11. An example of the type of images obtained in IVIS neutrophil imaging studies for *L. donovani* experiments.	14
13. Figure S12. The effect of vaccination on IL-10 and nitrite production by splenocytes from control or vaccinated mice infected with *L. donovani*	15
14. Figure S13. IFN-γ production by antigen and ConA stimulated splenocytes (A), IL-10 (B) and nitrite production (C) by splenocytes from control or vaccinated mice infected with *L. donovani*.	16

15. Figure S14. The effect of immunisation with different vaccine formulations on the local neutrophil and macrophage influx in control and vaccinated mice following infect with *L. donovani* 17
16. Figure S15. The effect of vaccination with different vaccines on immune responses and parasite burdens in mice infected with *L. donovani*. 18
17. Figure S16. The effect of vaccination on the amount of IFN-γ, IL-10 and nitrite produced by spleen cells from *L. donovani* infected mice. 19

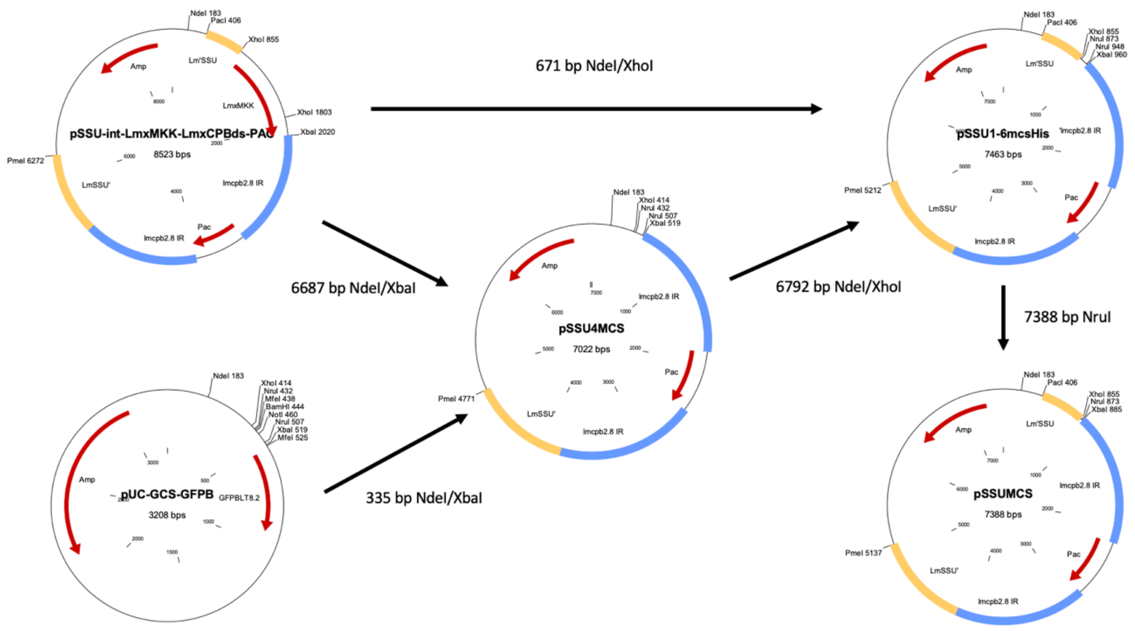

Figure S1. Schematic to generate the pSSUMCS intermediate plasmid for producing *L. tarentolae* promastigotes expressing γGCS from *L. donovani*, *L. major*, or *L. mexicana*. The pSSU-INT-lmmkk-lmcpbds-pac plasmid and pUC-GCS-GFPB plasmid were cleaved with the restriction enzymes NdeI and Xba. The 6687 bp and 335 bp DNA fragments produced were isolated and ligated to form the 7022 bp plasmid pSSU4MCS. Then pSSU-INT-lmmkk-lmcpbds-pac and pSSU4MCS were cleaved with the restriction enzymes NdeI and XhoI and the 671 bp and 6792 bp DNA fragments isolated and ligated to generate pSSU1-6mcsHIS (7463 bp). This plasmid was cleaved with NruI to isolate the 7388 bp fragment and religated to form the plasmid pSSUMCS.

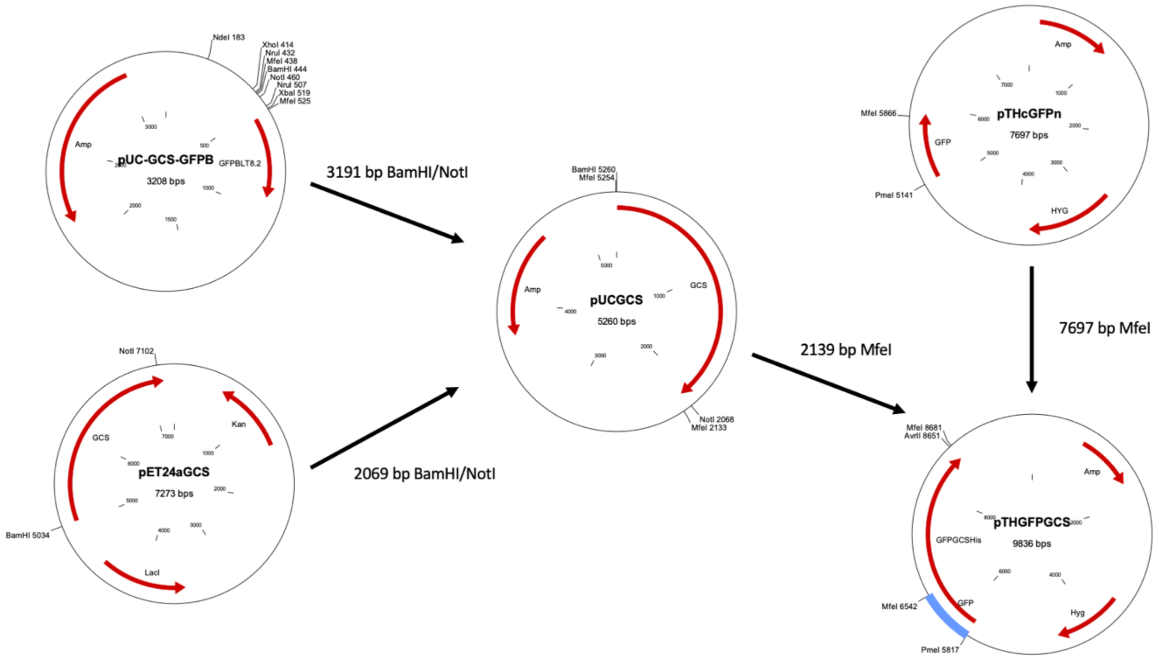

Figure S2. Schematic to generate the pTHGFPGCS intermediate plasmids used for producing *L. tarentolae* promastigotes expressing γGCS from *L. donovani*, *L. major*, or *L. mexicana*. The pUC-GCS-GFPB plasmid was cleaved with the restriction enzymes BamHI and NotI to isolate a 3191 bp DNA fragment. The *L. donovani*, *L. major* and *L. mexicana* γGCS expression plasmids pET24aLdonGCS, pET24aLmexGCS and pET24aLmajGCS were cleaved with the restriction enzymes BamHI and NotI. The resulting 2069 bp DNA fragments were isolated and ligated to the 3191 bp pUC-GCS-GFPB DNA fragment to result in the plasmids pUCLdonGCSHis, pUCLmexGCSHis and pUCLmajGCSHis. These three plasmids and pTHcGFPn were cleaved with the restriction enzyme MfeI to isolate the 2139 bp GCS DNA fragments of the three *Leishmania* species and a 7697 bp DNA fragment, which were ligated to form the pTHGFPGCS plasmids for the three *Leishmania* species.

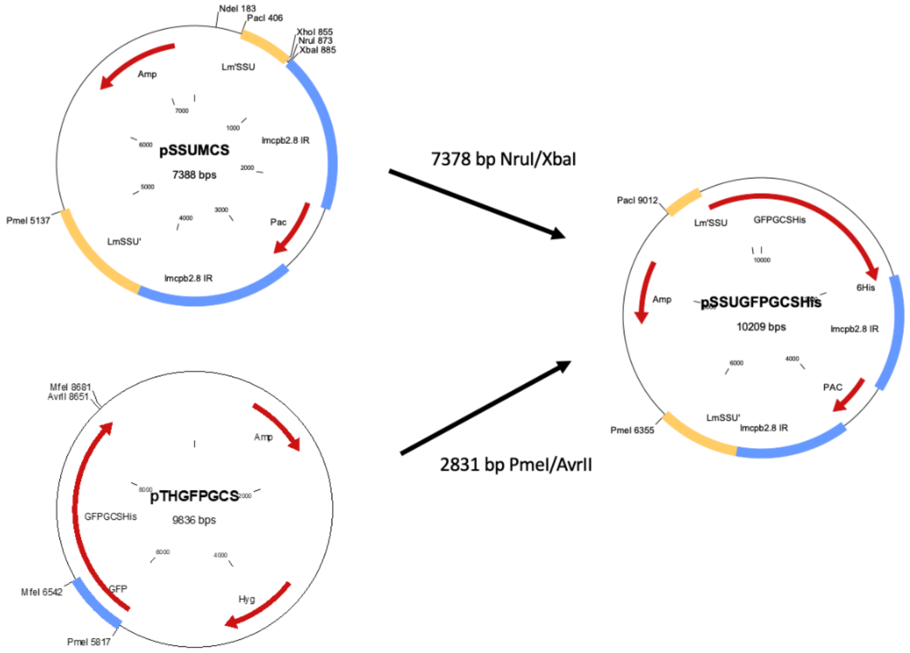

Figure S3. Schematic to generate pSSUGFPGCSHis plasmids used for producing *L. tarentolae* promastigotes expressing γGCS from *L. donovani*, *L. major*, and *L. mexicana*. The pTH plasmids (pTHGFPLdonGCSHis, pTHGFPLmexGCSHis and pTHGFPLmajGCSHis) were cleaved with the restriction enzymes PmeI and AvrII to generate the respective 2831 bp DNA fragment containing the GFPGCSHis sequences. The 7378 bp DNA fragment derived from pSSUMCS cleaved with NruI and XbaI and the 2831 DNA fragments were ligated to give the plasmids pSSUGFPLdonGCSHis, pSSUGFPLmexGCSHis, and pSSUGFPLmajGCSHis.

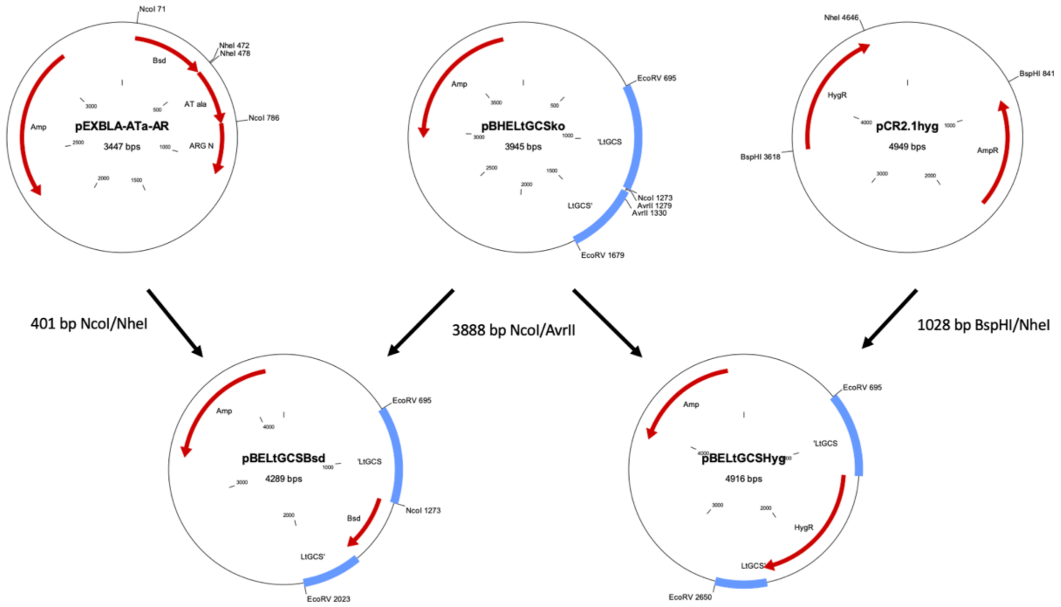

Figure S4. Schematic to generate the deletion constructs for *L. tarentolae* γGCS. The plasmids pEX-A2-BLA-AT-ArgN and pBEHLtGCSko were generated by gene synthesis (Biomatik, Ontario, Canada). The resistance marker genes for hygromycin B phosphotransferase and blasticidin-S-deaminase were isolated from pCR2.1Hyg and pEX-A2-BLA-AT-ArgN cut using BspHI/NheI and NcoI/NheI restriction enzymes, respectively. The resulting 1028 bp Hyg DNA-fragment and the 401 bp Bsd DNA-fragment were ligated with the 3888 bp DNA-fragment generated from pBEHLtGCSko, cleaved using the restriction enzymes NcoI and AvrII to produce the plasmids for deletion of *L. tarentolae* γGCS.

Table S1. The effect of vaccination on mean change in footpad size (mm ± SD) and mean number of amastigotes present in the footpad of *L. major* infected mice. Mice (n = 5/treatment) were immunised on days 0 and 14 with PBS alone (infection controls and uninfected controls) or vaccinated by subcutaneous injection of 1 × 10^7 *L. tarentolae* promastigotes (WT) or a 1:1:1 mixture of *L.t L.don* γGCS, *L.t L.maj* γGCS, *L.t L.mex* γGCS transgenic parasites (triple vaccine). On day 28 infection controls and vaccinated mice were infected with 1×10^7 *L. major* promastigotes by subcutaneous injection into the footpad (*Lmaj*Luc strain). Infected control and infected mice were sacrificed on day 56 and parasite burdens were determined by assessing footpad thickness of the infected footpad relative to the uninfected footpad over the course of infection. The experiment was terminated on day 59 post-infection i.e. day 73 of the experiment. **P* < 0.05, ***P* < 0.01 triple vaccine compared to infection control. [a]One of the mice the WT group was sacrificed before day 57 as it was likely to have a larger lesion than allowed by Home Office project licence restrictions, therefore this group contained 4 mice at these time points. NA – not applicable.

**Group**	**Day post-infection**					**Mean parasite number/mL ± SE ×10⁶** (Mean % reduction ± SE compared to control)
	**10**	**43**	**50**	**57**	**59**	
Control	0.04 ± 0.04	0.24 ± 0.11	0.31 ± 0.07	0.98 ± 0.22	0.93 ± 0.28	NA
WT	0.02 ± 0.01	0.45 ± 0.12	0.42 ± 0.13	0.41 ± 0.11[a]	0.55 ± 0.20[a]	26.6 ± 1.2
*L.t L.maj γ*GCS	0.07 ± 0.03	0.27 ± 0.06	0.76 ± 0.23	0.76 ± 0.23	0.82 ± 0.25	14.1± 3.81 (46% ± 0.15)
Triple	0.15 ± 0.06	0.19 ± 0.06	0.35 ± 0.21	0.35 ± 0.23*	0.49 ± 0.25*	0.70 ± 0.64** (94.31 ± 0.01)
Uninfected	0.09 ± 0.05	0.08 ± 0.06	0.0 ± 0.0	0.0 ± 0.0	0.05 ± 0.05	NA

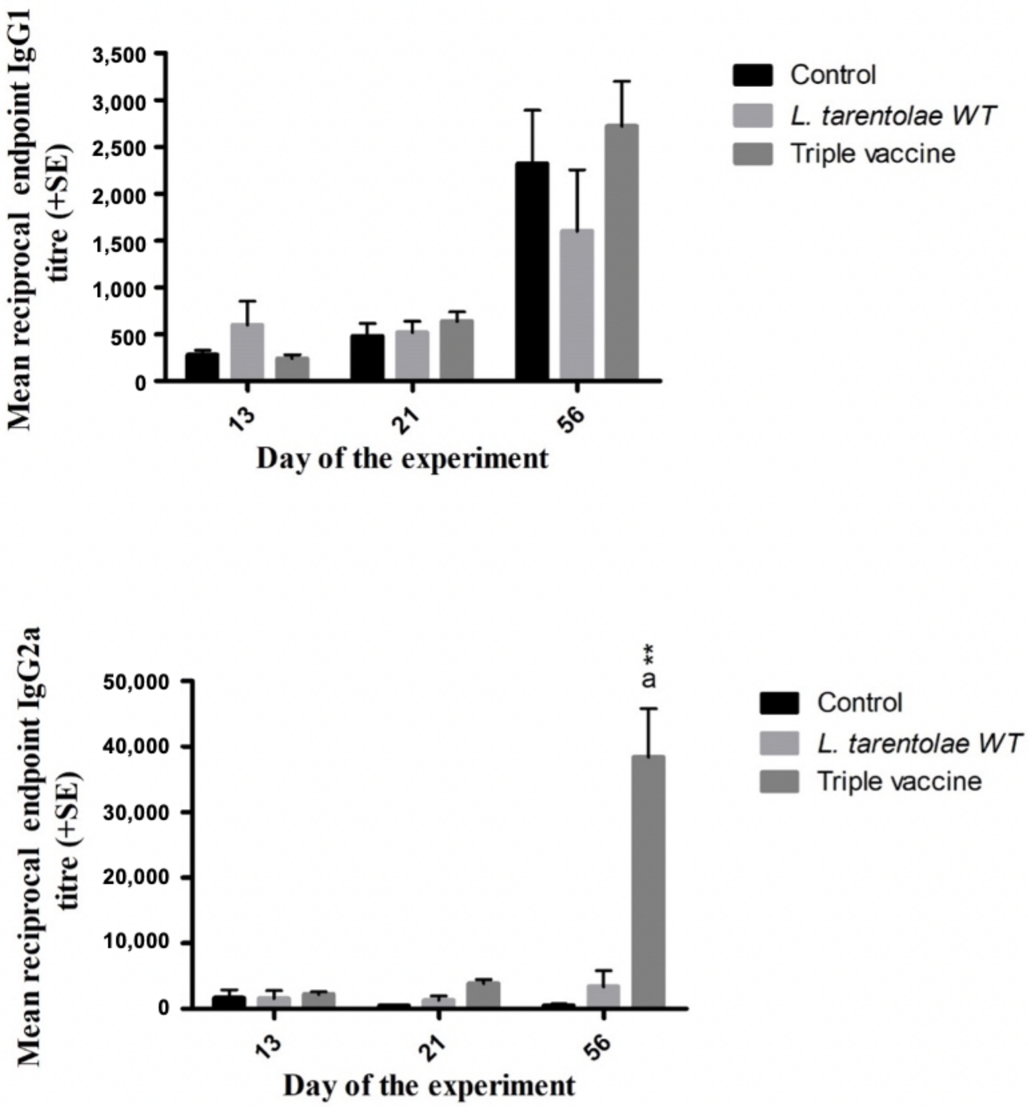

Figure S5. The effect of vaccination on the specific antibody response of mice of *L. major* infected mice. Mice (n = 5/treatment) were immunised on days 0 and 14 with PBS alone (infection controls) or vaccinated by subcutaneous injection of 1×10^7 *L. tarentolae* promastigotes (WT) or a 1:1:1 mixture of *L.t L.don* γGCS, *L.t L.maj* γGCS, *L.t L.mex* γGCS transgenic parasites (triple vaccine). On day 28 infection controls and vaccinated mice were infected with $1{\times}10^7$ *L. major* promastigotes by subcutaneous injection into the footpad (*Lmaj*Luc strain) and the experiment was terminated on day 59 post-infection i.e. day 73 of the experiment. Blood samples were taken from mice on day 13 (priming), day 27 (boost) and at sacrifice (day 59 post-infection) so that specific IgG1 and IgG2a antibody titres could be determined over the course of the experiment. **$P < 0.01$ triple vaccine compared to infection control, $^{a}P < 0.01$ compared to WT control.

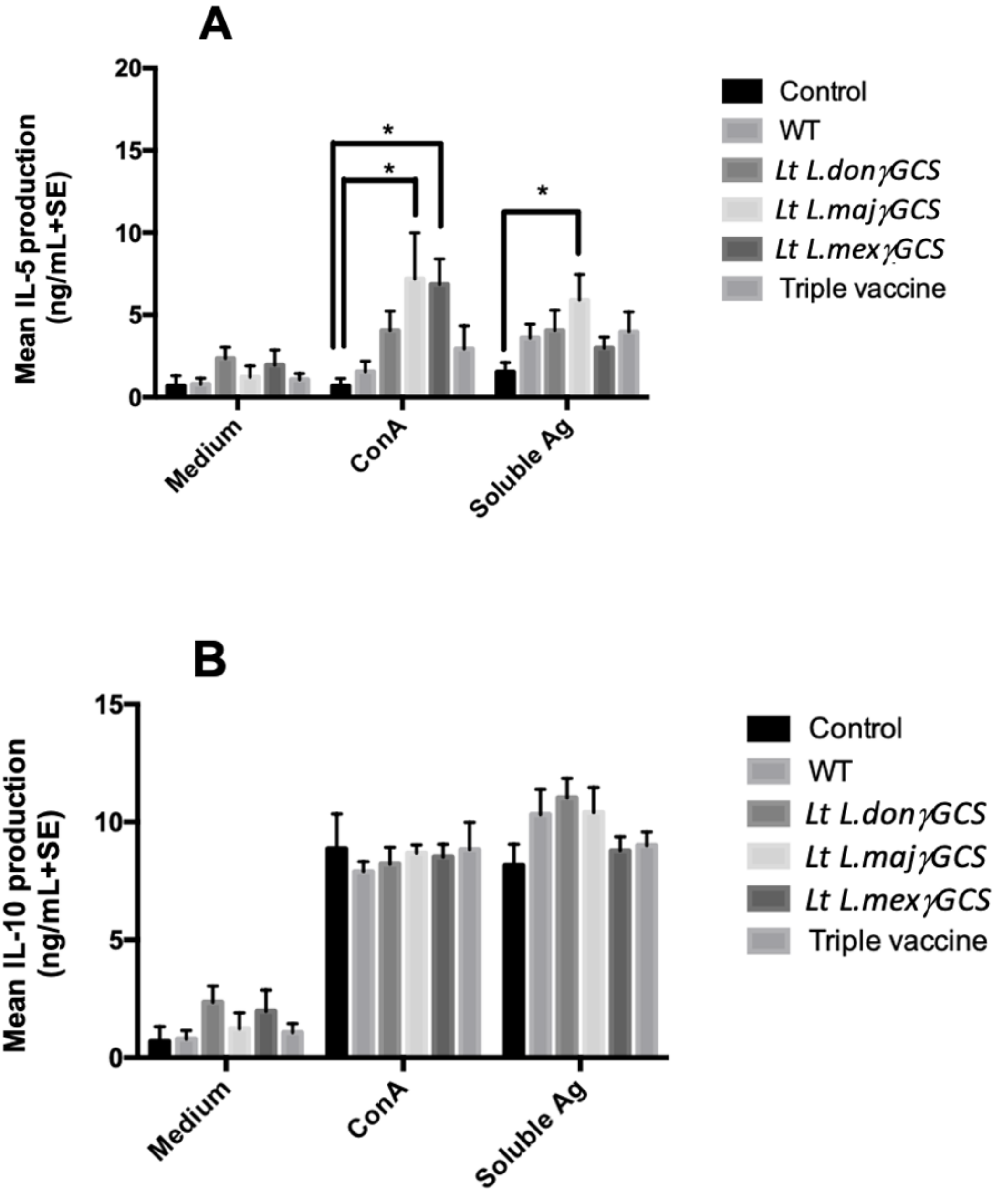

Figure S6. The effect of vaccination on the IL-5 (A) and IL-10 (B) production by splenocytes from control or vaccinated mice infected with *L. major* shown in Figure 5 in the main manuscript. Mice (n = 5/treatment) were immunised with PBS alone (control) or 1×10^7 *L. tarentolae* promastigotes (WT, *L.t L.don* γGCS, *L.t L.maj* γGCS, *L.t L.mex* γGCS alone or a mixture of all three [1:1:1 mixture, triple vaccine] on days 0 and 14. On day 28 control and immunised mice were infected with 1×10^7 *L. major* promastigotes by subcutaneous injection and then mice were sacrificed on day 56. Splenocytes (5×10^5/mL) from the mice were incubated with medium alone (controls), ConA (5 μg/mL) or *L. major* soluble antigen (25 μg/mL) for 72 hr and the mean IL-5 (A) or IL-10 (B) production determined by ELISA. **P* < 0.05 for the groups compared.

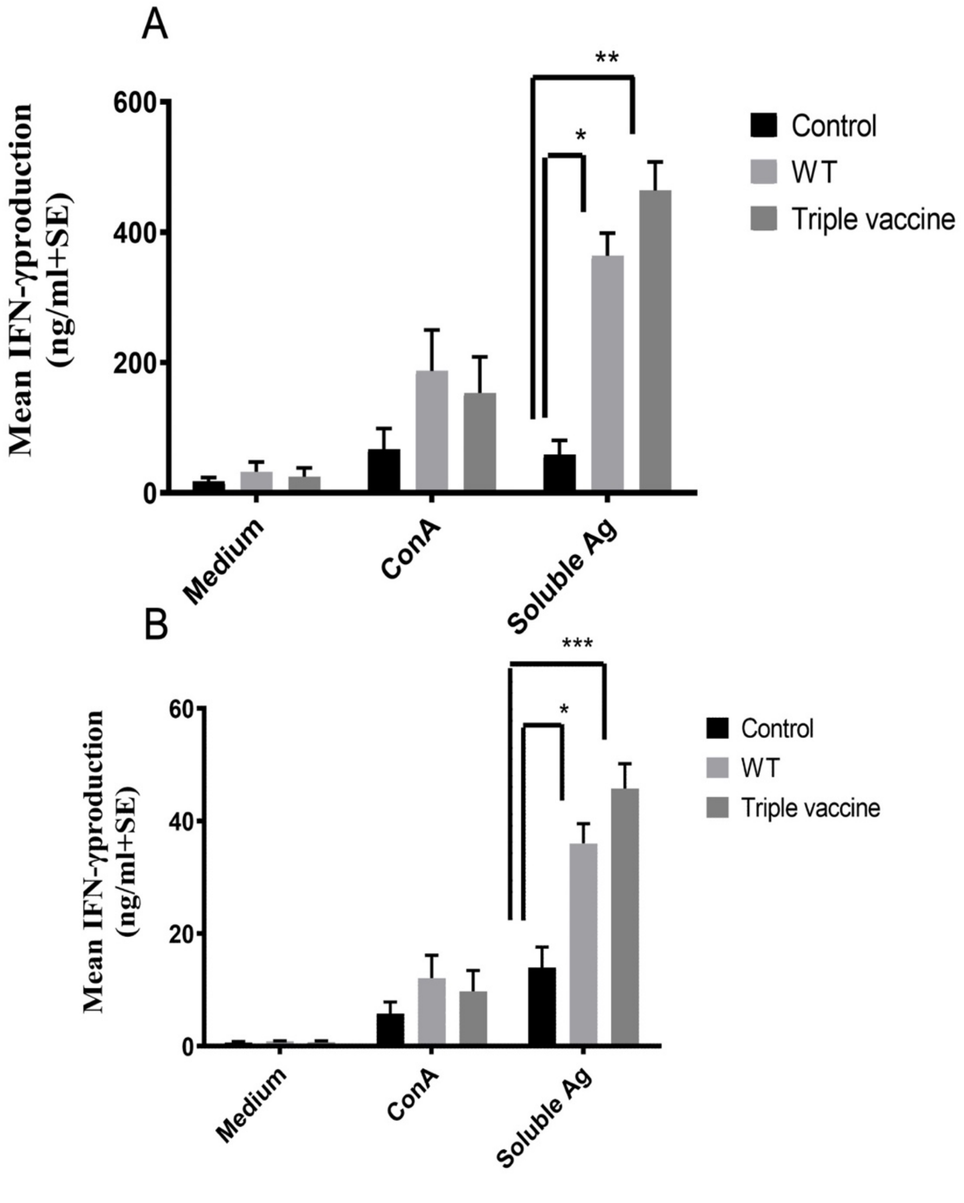

Figure S7. The effect of vaccination on the mean IFN-γ production of popliteal lymph node cells (A) or spleen (B) from *L. major* infected mice shown in Table S1. Cells (5 x 10^5/well) were stimulated with medium alone (controls), specific antigen (25 μg/ml) or ConA (5 μg/ml) for 72 hours and then samples were stored at -20°C until IFN-γ levels could be determined using an ELISA assay. *$P < 0.05$, **$P < 0.01$, ***$P < 0.001$ for groups shown.

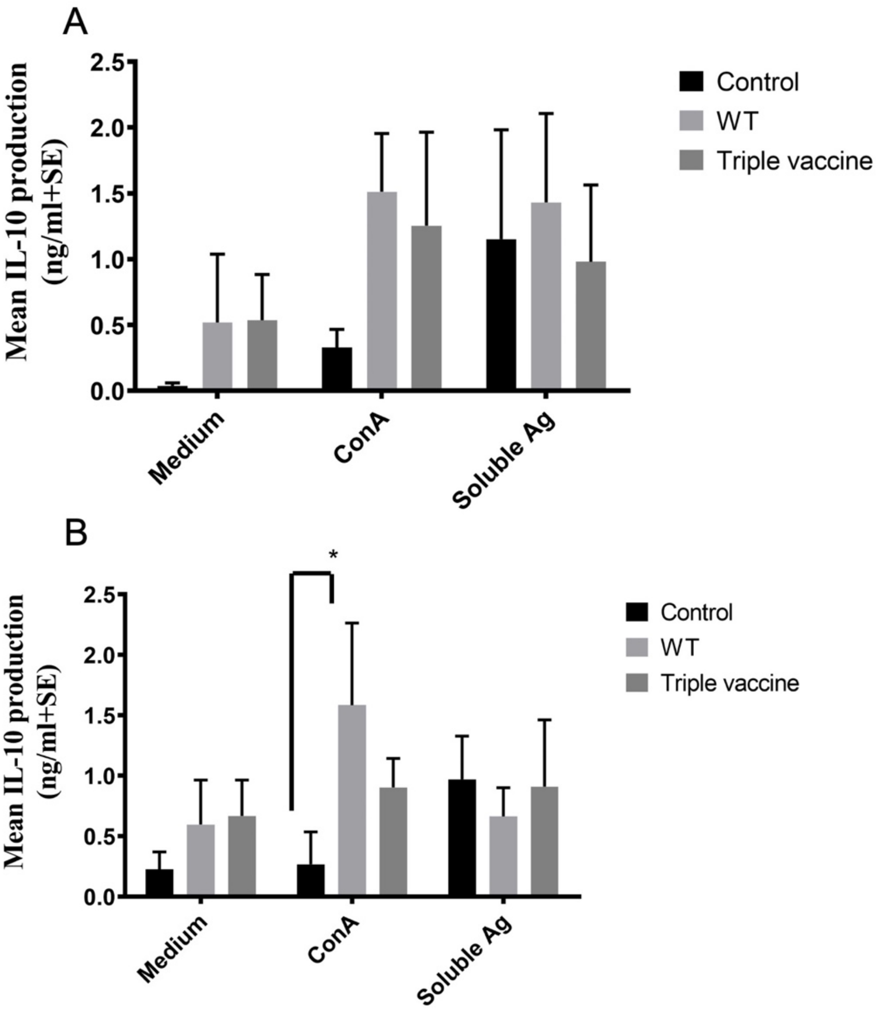

Figure S8. The effect of vaccination on the mean IL-10 production of popliteal lymph node cells (A) or spleen (B) from *L. major* infected mice shown in Figure 5 in the main manuscript. Cells (5 x 10^5/well) were stimulated with medium alone (controls), specific antigen (25 μg/ml) or ConA (5 μg/ml) for 72 hours and then samples were stored at -20°C until IL-10 levels could be determined using an ELISA assay. *$P < 0.05$, **$P < 0.01$, ***$P < 0.001$ for groups shown.

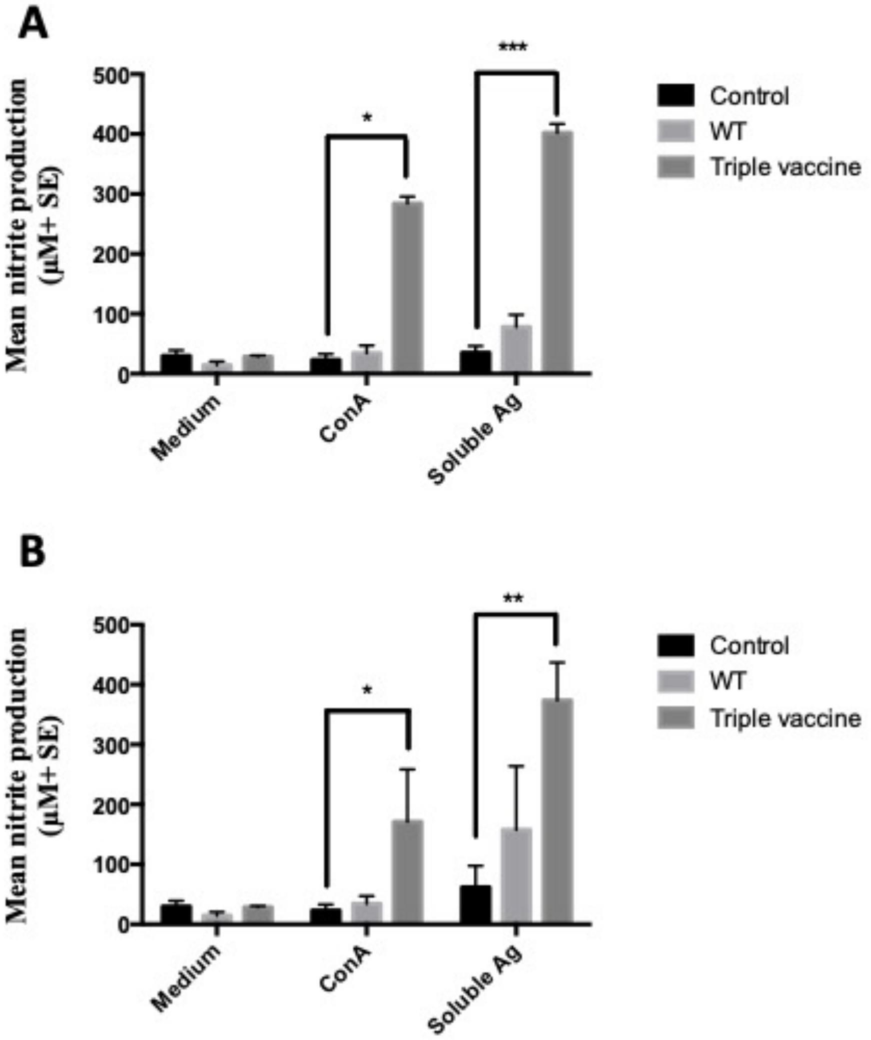

Figure S9. The effect of vaccination on the mean nitrite production of popliteal lymph node cells (A) or spleen (B) from *L. major* infected mice shown in Table S1. Cells (5 x 10^5/well) were stimulated with medium alone (controls), specific antigen (25 µg/ml) or ConA (5 µg/ml) for 72 hours and then samples were stored at -20°C until nitrite levels could be determined using a Greiss assay. *$P < 0.05$, **$P < 0.01$, ***$P < 0.001$ for groups shown.

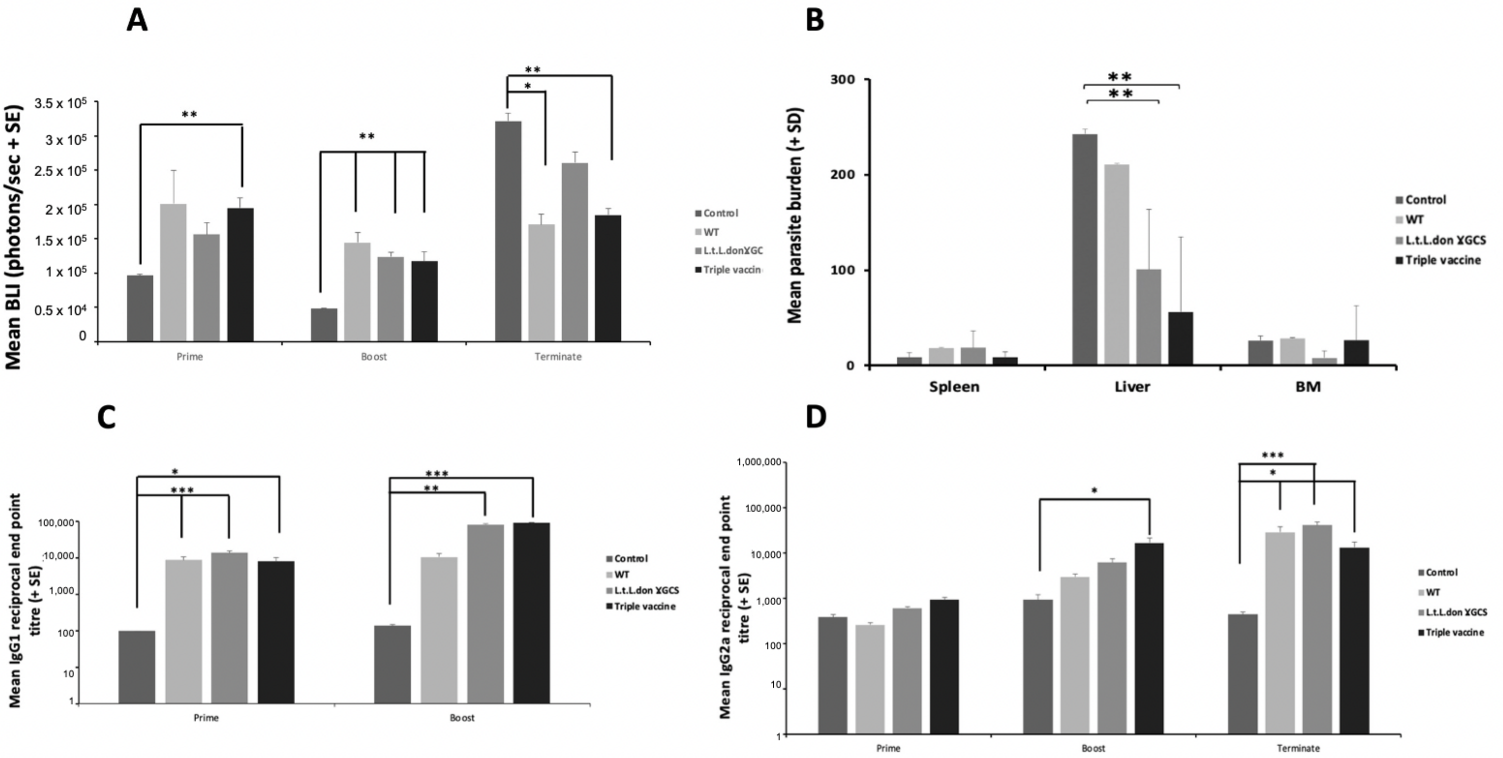

Figure S10. The effect of vaccination with different vaccines on neutrophil influx, antibody responses and parasite burdens of mice infected with *L. donovani*. Mice (n = 5/treatment) were immunised on days 0 and 21 with PBS alone (control), 1 × 10^7 *L. tarentolae* promastigotes (WT), 1 × 10^7 *L.t L.don* γGCS promastigotes (*L.t L.don* γGCS) or a 1:1:1 mixture of *L.t L.don* γGCS, *L.t L.maj* γGCS, *L.t L.mex* γGCS transgenic parasites (1 × $10^{7,}$ triple vaccine). The effect of vaccination on neutrophil recruitment 3 hours post-treatment, after injecting mice with luminol solution (150 mg/kg) and determining the amount of bioluminescence (mean bioluminescence, total flux, p/s) present at the injection site (A). Mice were infected on day 42 with 2 × 10^7 *L. donovani* amastigotes and parasite burdens were determined on day 56 (B). Specific *L. donovani* IgG1 (C) and IgG2a (D) antibody titres were determined over the course of the study. *$P < 0.05$, **$P < 0.001$, ***$P < 0.0001$ compared to control.

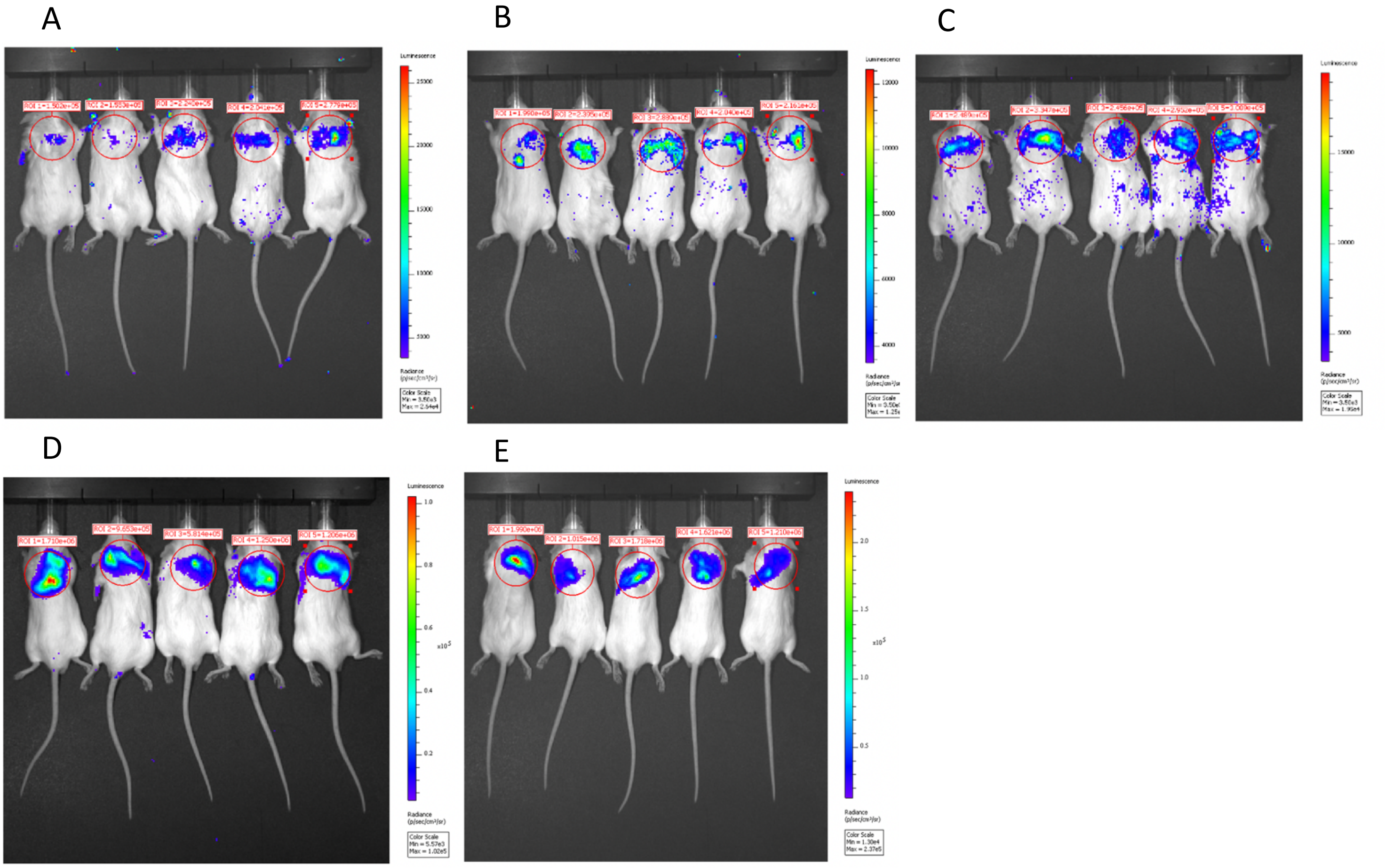

Figure S11. An example of the type of images obtained in IVIS neutrophil imaging studies for *L. donovani* experiments**.** In studies the same size region of interest was used for every reading in an experiment so that the same sized area was used to collect data. BALB/c mice (n = 5/treatment) were immunised on days 0 and 21 with PBS alone (control, A), PODS-Empty (50 million/mouse, B), PODS-IL-2 (50 million/mouse, C), 1 × 10^7 *L.t L.don* γGCS promastigotes alone (vaccine, D) or mixed with PODS-IL-2 (50 million/mouse, vaccine, E). The amount of bioluminescence emitted from the injection site (mean bioluminescence, total flux, p/s) was determined 3 hours after the first immunisation, mice were given 0.2 ml luminol solution (150 mg/mL, PBS) by intraperitoneal injection and imaged for 2 mins starting from 5 minutes after injection.

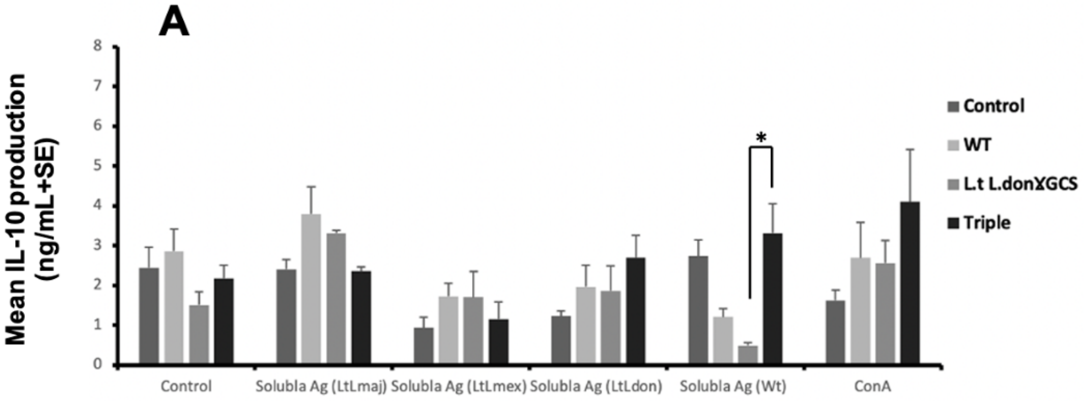

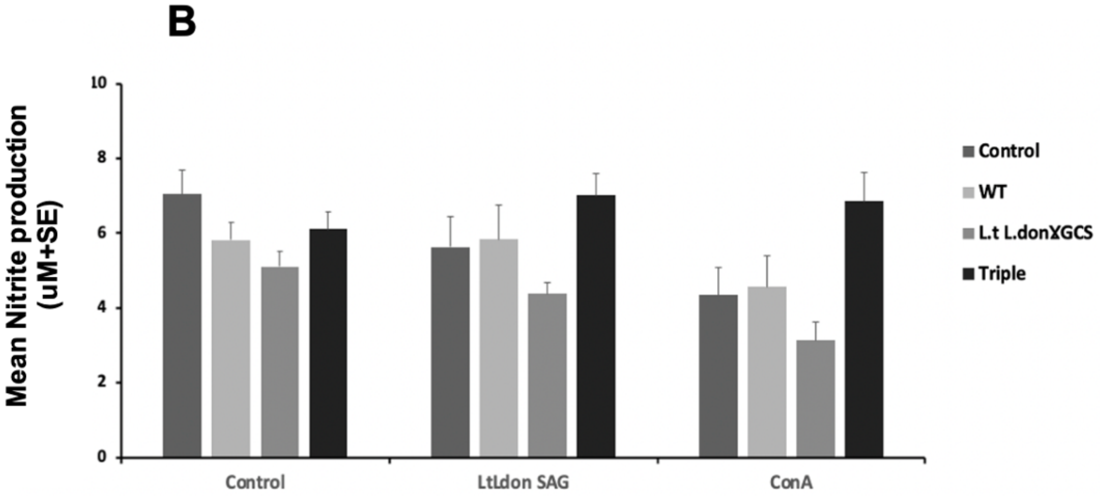

Figure S12. The effect of vaccination on IL-10 and nitrite production by splenocytes from control or vaccinated mice infected with *L. donovani*. Mice (n = 5/treatment) were immunised on days 0 and 21 with PBS alone (control) or 1 × 10^7 *L. tarentolae* promastigotes (WT), *L.t L.don* γGCS transgenic parasites (Lt L.donγGCS) or a 1:1:1 mixture of *L.t L.don* γGCS, *L.t L.maj* γGCS, *L.t L.mex* γGCS transgenic parasites (1 × 10^7, triple vaccine). On day 42 mice were infected with 2 × 10^7 *L. donovani* amastigotes and on day 56 the study was terminated. Splenocytes (5 × 10^5/mL) from each mouse were incubated with medium alone (controls), ConA (5 µg/mL) or *L. donovani* soluble antigen (50 µg/mL) for 72 h and the amount of IL-10 present in cell supernatants determined by ELISA (A); the amount of nitrite was determined using a Greiss assay (B). **P* < 0.05 for the groups compared.

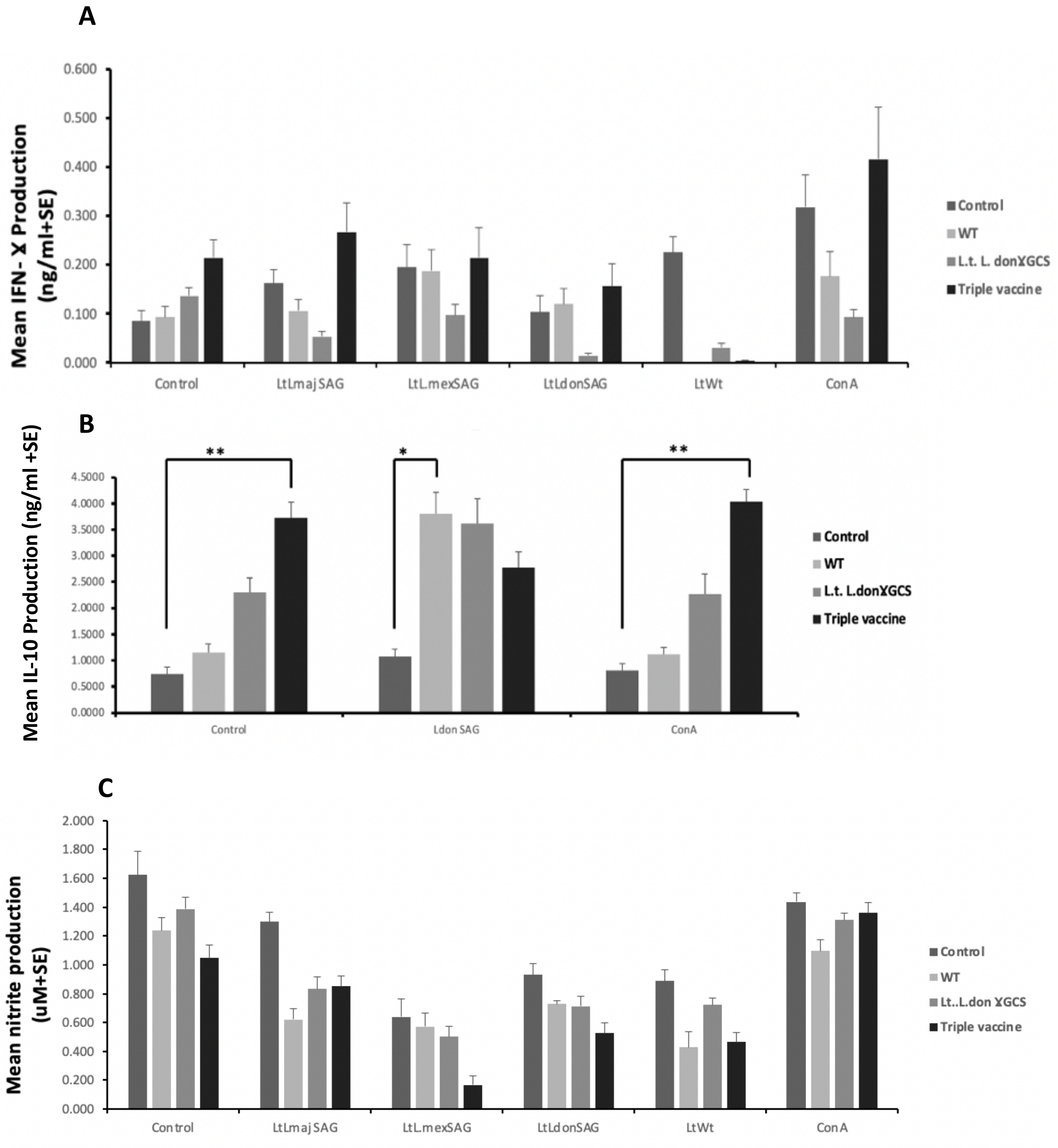

Figure S13. IFN-γ production by antigen and ConA stimulated splenocytes (A), IL-10 (B) and nitrite production (C) by splenocytes from control or vaccinated mice infected with *L. donovani*. Mice (n = 5/treatment) were immunised on days 0 and 21 with PBS alone (control) or 1 × 10^7 *L. tarentolae* promastigotes (WT) *L.t L.don* γGCS) or a 1:1:1 mixture of *L.t L.don* γGCS, *L.t L.maj* γGCS, *L.t L.mex* γGCS transgenic parasites (1 × $10^{7,}$ triple vaccine). On day 42, the mice were infected by intravenous injection with 2 × 10^7 *L. donovani* amastigotes and sacrificed on day 56. Splenocytes (5 × 10^5/mL) from each mouse were incubated with medium alone (controls), ConA (5 μg/mL) or soluble antigen (50 μg/mL) for 72 hr and cytokines amount of present in cell supernatants determined by ELISA and the amount of nitrite was determined using a Griess assay. *$P < 0.05$, **$P < 0.001$ compared to control.

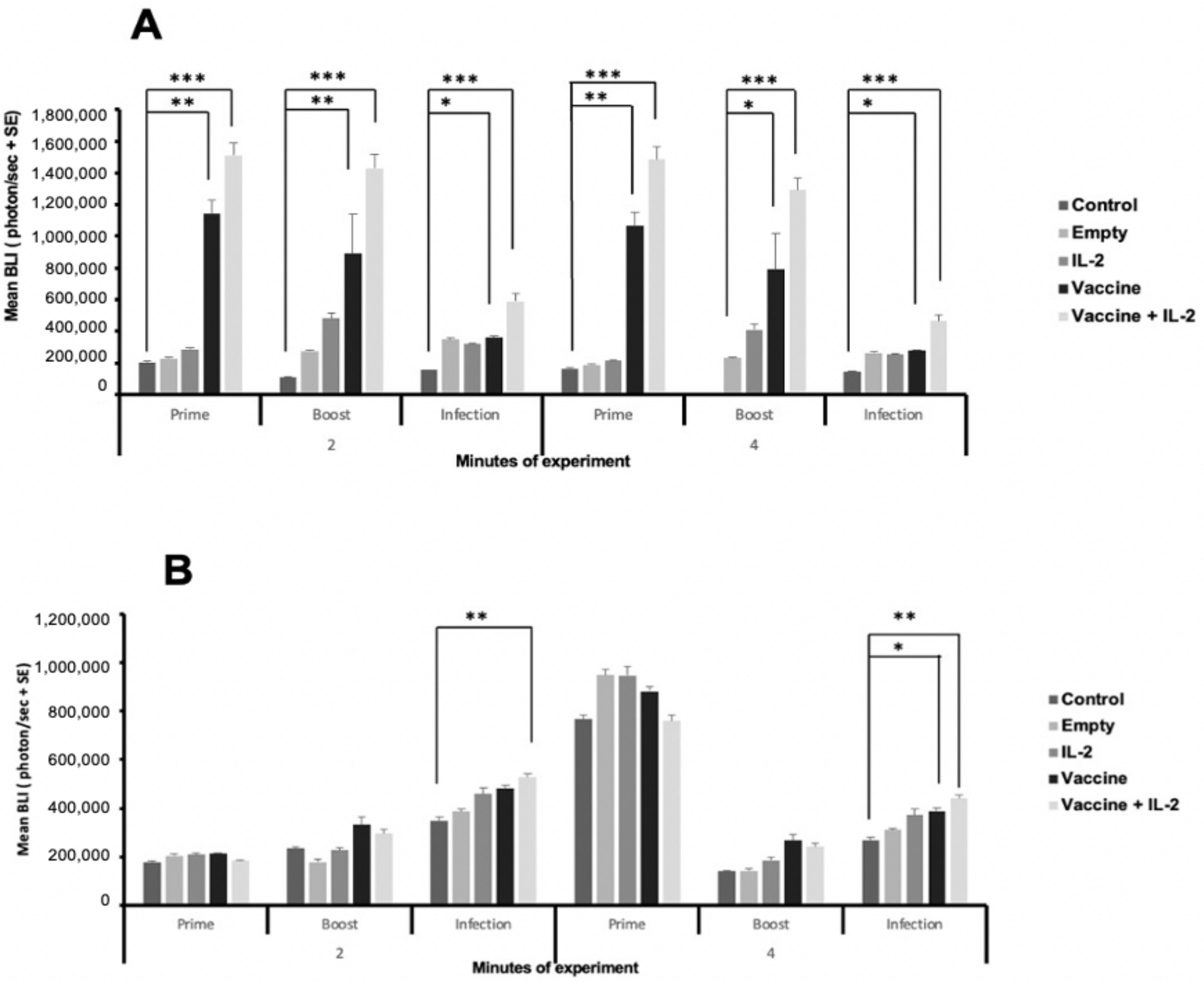

Figure S14. The effect of immunisation with different vaccine formulations on the local neutrophil and macrophage influx in control and vaccinated mice following infection with *L. donovani*. Mice (n = 5/treatment) were immunised on days 0 and 21 with PBS alone (control), PODS-Empty (Empty), PODS-IL-2 (IL-2), 1 × 10^7 *L.t L.don* γGCS promastigotes alone (vaccine) or 1 × 10^7 *L.t L.don* γGCS promastigotes mixed with PODS-IL-2 (vaccine + IL-2). On day 42, the mice were infected by intravenous injection with 2 × 10^7 *L. donovani* amastigotes. Three hours after infection with *L. donovani* each mouse was injected with luminol solution (150 mg/mL, saline) and the effect on neutrophil recruitment amount assessed by determining the amount of bioluminescence (Mean BLI, total flux, p/s) emitted from the visceral area of the mice determined using IVIS imaging (A). The same mice were was injected with lucigenin solution (10 mg/kg, saline) 72 hours after infection with *L. donovani* and macrophage recruitment (B) assessed by determining the amount of bioluminescence emitted from the visceral area of the mice determined using IVIS imaging. *$P < 0.05$, ** $P < 0.01$, *** $P < 0.001$.

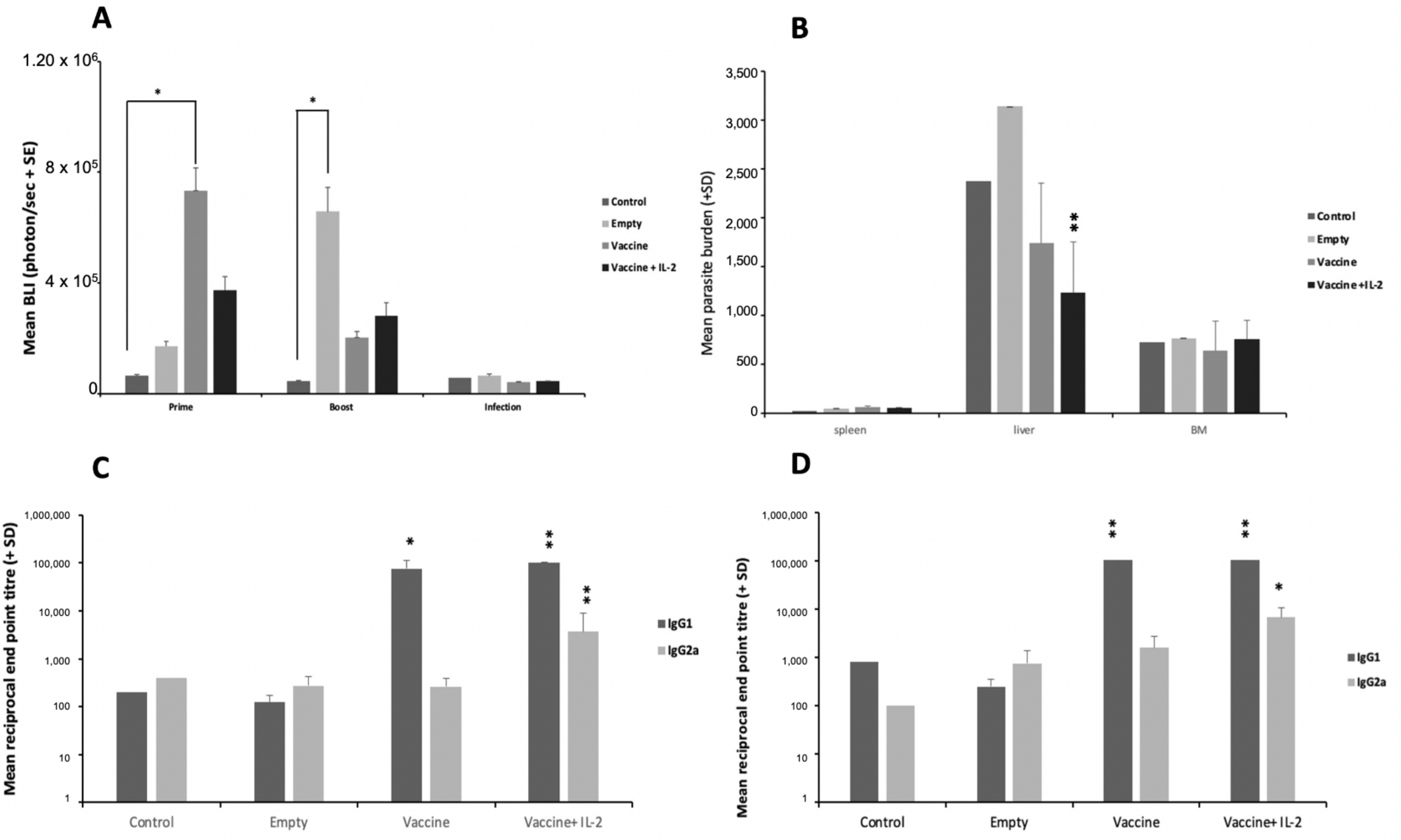

Figure S15 The effect of vaccination with different vaccines on parasite burdens and the immune responses of *L. donovani* infected mice. BALB/c mice (n = 5/treatment) were immunised on days 0 and 21 with PBS alone (control), PODS-Empty (50 million/mouse), 1×10^7 *L.t L.don* γGCS promastigotes alone (vaccine) or 1×10^7 *L.t L.don* γGCS promastigotes mixed with PODS-IL-2 (50 million/mouse, vaccine + IL-2). The effect of vaccination on neutrophil recruitment 3 hours post-treatment, after injecting mice with luminol solution (150 mg/kg) and determining the amount of bioluminescence (Mean BLI, total flux, p/s) present at the injection site (A). Mice were infected on day 42 with 2×10^7 *L. donovani* amastigotes and parasite burdens were determined on day 56 (B). The specific *L. donovani* antibody of mice on day 42 (C) and day 56 (D) are shown. $^*P < 0.05$, $^{**}P < 0.001$ compared to control.

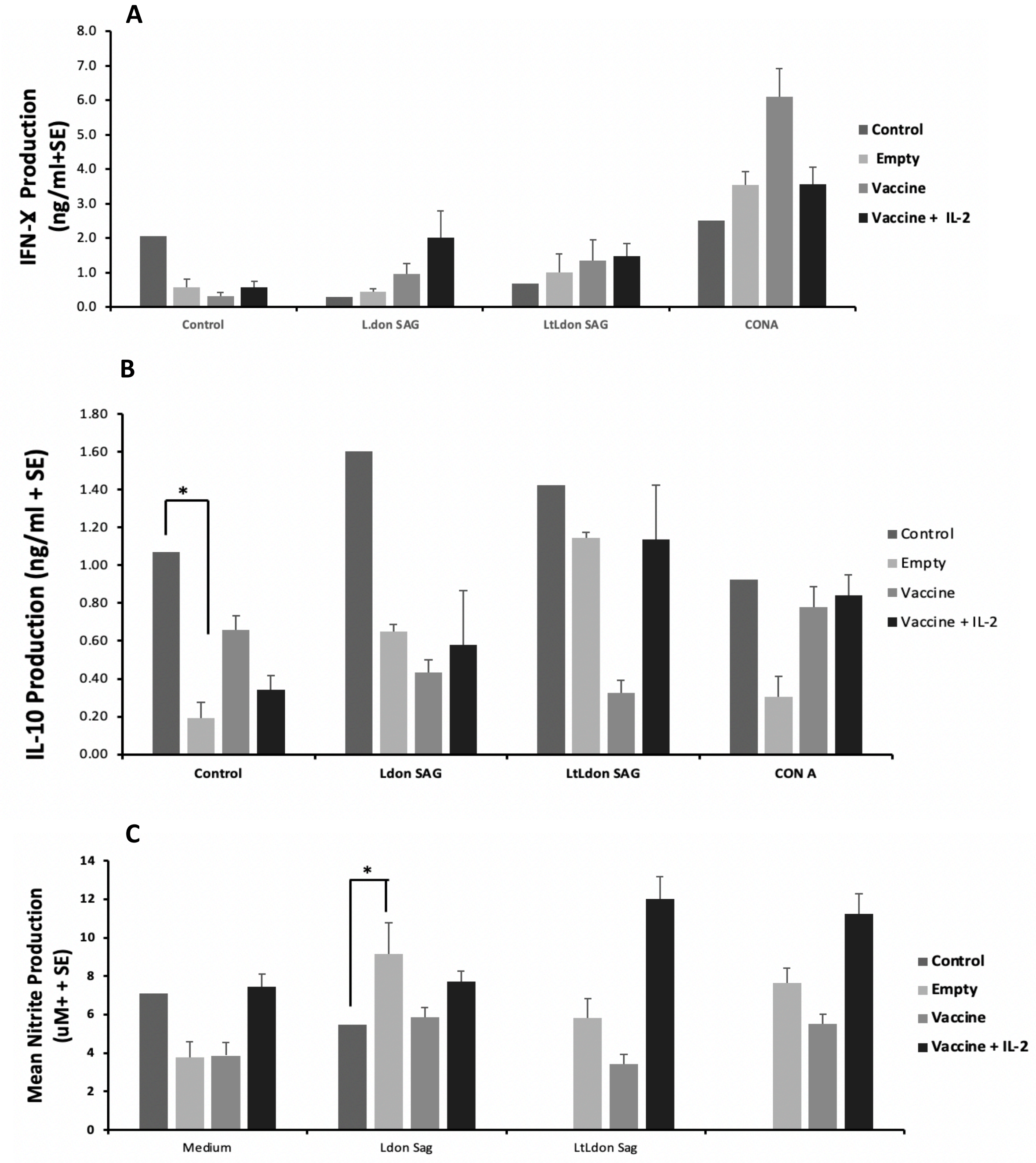

Figure S16 The effect of vaccination on the amount of IFN-γ, IL-10 and nitrite produced by spleen cells from *L. donovani* infected mice. BALB/c mice (n = 5/treatment) were immunised on days 0 and 21 with PBS alone (control), PODS-Empty (50 million/mouse), 1×10^7 *L.t L.don* γGCS promastigotes alone (vaccine) or 1×10^7 *L.t L.don* γGCS promastigotes mixed with PODS-IL-2 (50 million/mouse, vaccine + IL-2). On day 42, the mice were infected by intravenous injection with 2×10^7 *L. donovani* amastigotes and sacrificed on day 56. Splenocytes (5×10^5/mL) from each mouse were incubated with medium alone (controls), ConA (5 μg/mL) or soluble antigen (50 μg/mL) for 72 hr. The amount of amount of IFN-γ (A) and IL-10 (B) produced by cells was determined by ELISA and the amount of nitrite (C) produced by cells was determined using a Griess assay. *$P < 0.05$ for groups indicated.
